# Community activism as a strategy to reduce intimate partner violence (IPV) in rural Rwanda: Results of a community randomised trial

**DOI:** 10.7189/jogh.10.010406

**Published:** 2020-06

**Authors:** Sangeeta Chatterji, Erin Stern, Kristin Dunkle, Lori Heise

**Affiliations:** 1 Johns Hopkins Bloomberg School of Public Health, Johns Hopkins University, Baltimore, Maryland, USA; 2 Gender and Health Research Unit, South African Medical Research Council, Cape Town, South Af; 4 Johns Hopkins School of Nursing, Johns Hopkins University, Baltimore, Maryland, USA; 5 London School of Hygiene and Tropical Medicine, London, UK (affiliation at start of project)

## Abstract

**Background:**

There is considerable interest in community organising and activism as a strategy to shift patriarchal gender norms, attitudes and beliefs and thus reduce intimate partner violence (IPV). Yet there is limited insight into how activism actually translates into reduced violence, including how aspects of programme implementation or cultural context may affect impact. This study evaluates the community activism/mobilisation portion of *Indashyikirw*a, a multi-component, IPV prevention programme implemented in rural Rwanda. The activism part of *Indashyikirwa* was based on *SASA!*, a promising program model from Uganda with demonstrated effectiveness.

**Methods:**

We implemented two separate cross-sectional surveys as part of a larger community randomised controlled trial to assess the impact of the community portion of *Indashyikirwa* on preventing physical and/or sexual IPV and other secondary outcomes at a community level. The survey consisted of a random household-based sample of 1400 women and 1400 men at both waves. Surveys were conducted before community-level activities commenced and were repeated 24 months later with a new cross-sectional sample. Longitudinal, qualitative data were collected as part of an embedded process evaluation.

**Results:**

There was no evidence of an intervention effect at a community level on any of the trial’s primary or secondary outcomes, most notably women’s experience of physical and/or sexual IPV from a current male partner in the past 12 months (adjusted odds ratio (aOR) = 1.25; 95% confidence interval (CI) = 0.92-1.70, *P* = 0.16), or men’s perpetration of male-to-female physical and/or sexual IPV (aOR = 1.02; 95% CI = 0.72-1.45, *P* = 0.89). Process evaluation data suggest that delays due to challenges in adapting and implementing *SASA!*-style activites in rural Rwanda may account for the trial’s failure to measure an effect. Additionally, the intervention strategy of informal activism was not well suited to the Rwandan context and required considerable modification.

**Conclusions:**

Failure to reduce violence when implementing an adaptation of *SASA!* in rural Rwanda highlights the importance of allowing sufficient time for adapting evidence-based programming (EBP) to ensure cultural appropriateness and fidelity. This evaluation held little chance of demonstrating impact since the project timeline forced endline evaluation only months after certain elements of the programme became operational. Donors must anticipate longer time horizons (5 to 7 years) when contemplating evaluations of novel or newly-adapted programmess for reducing IPV at a population level. These findings also reinforce the value of including embedded process evaluations when investing in rigorous trials of complex phenomena such as community activism.

**Trial registration:**

ClinicalTrials.gov, NCT03477877

Intimate partner violence (IPV) is a critical global health issue that affects 30% of women worldwide [1]. Emerging evidence affirms that levels of IPV can be reduced and suggests several promising areas for intervention. Community activism to shift the patriarchal norms, attitudes and beliefs that undergird IPV is one strategy that has garnered considerable attention in recent years. Rigorous trials in sub-Saharan Africa have demonstrated that community activism, mobilisation, and organised diffusion can be effective in reducing IPV [2-5]. For example, *SASA!* and *SHARE*, both community mobilisation programs implemented in Uganda, documented reductions in women’s experiences of IPV over 4 years as well as shifts in the attitudes, beliefs, and social norms perceived to perpetuate such violence [3,6,7]. Importantly, other mobilisation and community activism interventions have found no such impact on IPV [8,9], suggesting that implementation and context are crucial for achieving gains in safety and well-being at the community level.

Moreover, there is limited understanding of how activism actually translates into reduced violence, including how cultural context and programme implementation may affect impact. Process evaluations have uncovered unforeseen challenges such as lack of institutional support for staff and activist volunteers [8] and individual factors such as illness, family, and economic pressures that have negatively impacted the success of some mobilisation interventions [10]. Trials of community-based IPV interventions remain scarce given the high costs and methodological challenges of evaluating complex interventions designed to catalyse widespread social change and shift harmful social norms [11,12].

In this paper, we report on an impact evaluation of a large-scale community activism/mobilisation programme implemented as part of a multi-component intervention to reduce IPV in rural Rwanda. The programme, known as *Indashyikirwa* (“Agents of Change” in Kinyarwanda, trial identifier: NCT03477877), was implemented by CARE International, Rwanda; the Rwanda Women’s Network (RWN); and the Rwanda Men’s Resource Centre (RWAMREC) between 2015 and 2018, with funding from the Rwandan office of the Department for International Development (DFID-R). The full *Indashyikirwa* program included four interlocking components: a 21-session couples’ curriculum; community outreach by trained community activists; the creation of an enabling environment through training and active involvement of key opinion leaders; and provision of support to victims through the creation of women’s “safe spaces” [13,14].

This paper reports exclusively on the evaluation of the community activism portion of the intervention, conducted by external researchers funded by DFID UK as part of a separate initiative known as What Works to Prevent Violence Against Women and Girls. The findings reported herein derive from two repeat cross-sectional surveys of community members, conducted as part of a larger community randomised trial, with qualitative data from a nested process evaluation [15]. A separate manuscript reports on the impact that the couple’s curriculum had on relationship dynamics and IPV levels among couples who participated in the programme’s 5-month couples’ curriculum (for more details on the couple’s curriculum, see [13]). Longitudinal evaluation of the couples’ programme demonstrated that couples who participated in the training reported less physical, sexual, emotional, and economic IPV; improved relationship quality; better communication; improved conflict management; fewer depressive symptoms; better oveall health; and attitudes less supportive of wife beating, than did control couples at 12 and 24 months followup [16]. This paper reports on the community-wide elements of *Indashyikirw*a including activist activities, implementation of women’s safe spaces and training of local opinion leaders. We assess the extent to which these activities impacted community-level experience and perpetration of physical and/or sexual violence, attitudes towards violence, support for survivors and how the results relate to the design and implementation of the intervention.

## Background and intervention design

*Indashyikirwa* was implemented in seven districts in the Eastern, Northern and Western provinces of Rwanda, in predominantly rural, widely-dispersed communities. Although the Rwandan government enacted the Prevention and Punishment of Gender-Based Violence Law in 2008, IPV is a persistent phenomenon in Rwanda, as in many other settings. According to the 2014-15 Rwanda Demographic and Health Survey, 34% of women aged 15 to 49 in the general population experienced physical and/or sexual violence by a husband/partner in the past 12 months [17].

It was against this backdrop that CARE Rwanda, together with RWAMREC and RWN, sought to design and implement an evidence-informed programme to reduce violence within intimate partnerships in Rwanda. The original idea was to build from the experiences of the partner organisations and from insights generated from an earlier evaluation of *SASA!*, a programme designed by the Ugandan NGO, Raising Voices, to reduce HIV and IPV [7,18]. Raising Voices, together with the local CBO, CEDOVIP, successfully reduced the population prevalence of current sexual and/or physical IPV by 52% (a 16 pp absolute decrease in physical IPV) in a high density area of Kampala over 4 years [7].

**Table 1** briefly summarises the original *SASA!* programme, and notes how *Indashyikirwa* compares with *SASA!* [13]. In the results and discussion sections of this paper, we further explore ways that *Indashyikirwa*, as implementated, departed from the *SASA!* model and from *indashyikirwa’s* original design.

**Table 1:** Comparison of *Indashykirwa* and *SASA!*

Programme	*SASA!* model by Raising Voices	*Indashyikirwa* as implemented in Rwanda
Location of Intervention	Kampala, Uganda (urban informal settlement)	14 districts across rural Rwanda; trainings and coordination meetings took place centrally with activists doing outreach in disparate villages
Components of Intervention	Four strategies (communication materials, media and advocacy, local activism and training) implemented over four phases. Each phase focuses on a different outcome: Start (knowledge), Awareness (attitude), Support (skills), Action (behavior).	21 session couples’ curriculum focuses on gender, power, relationship skills, triggers of violence, harmful alcohol use, sexuality, etc.; 16 additional sessions on activism skills with a sub-set of couples trained as community activists (CAs); Activities undertaken at village level by trained CAs; Creation and staffing of women’s safe spaces; Engagement of opinion leaders through training and ongoing coordination meetings
Approach	Community activists (CAs) encourage reflection and promote action on power and violence through informal engagement with community members, relying on “quick chats,” games, drama, and creative communication materials and techniques, rather than on workshops, or speaking at formal public events (SASA Fidelity Brief)	Activists primarily conducted activities at more formal venues, including at village savings and loans meetings, community meetings, *umuganda* days, domestic violence committees, and parents evening forums, although this shifted to more informal settings by the end of the programme
Phasing	The **Start Phase** nurtures one’s ‘power within,’ the **Awareness Phase** deepens analysis of men’s ‘power over’ women and the communities silence about this, the **Support Phase** fosters joining ‘power with’ others, and **Action Phase** encourages the use of ‘power to’ make and sustain positive change	Trainings with couples, community activists, opinion leaders and women’s safe space facilitators included elements from all phases; For the community activism component, the **Start** and **Action phases**, and **Support and Awareness phases** were merged given that adaptation-related needs and processes took longer than originally expected
Duration of Intervention	3-5 years to complete all phases of activism activities	1 year for inception and design of intervention; 9 mo establishment of women’s safe spaces; training of opinion leaders and couples curriculum; 3 mo selection and training of activists; 18 mo for activism led by sub-set of couples and operation of women’s safe spaces (see timeline)
Preparation of staff and activists	8 hours of training at the beginning of each phase	21 sessions of 3 hours each for couples; 2 weeks of 16 additional sessions on activism
Field Officers per Activist	At least one dedicated staff member to regularly support and mentor twenty-five community activists (SASA Fidelity Brief)	One RWN staff member for every twenty-two women’s safe space facilitators and one RWAMREC staff member for every forty community activists
Implementation of Phased Programming	Monitoring and evaluation tools assess progress at each phase and determine readiness for the subsequent phase	Trainings with couples, community activists, opinion leaders and women’s safe space facilitators included elements from all phases
Activism Tools and Strategies	The Communication Materials strategy include creative and positive materials, such as posters, comics and info-sheets, to support community members to think and talk about power and violence against women. The Local Activism strategy includes initiatives that create informal opportunities for personal reflection, critical thinking and public dialogue about power and violence against women. The Media & Advocacy strategy aims to influence public priorities, by making violence against women a popular media topic and by engaging local leaders, policymakers and journalists and includes soap operas, operas, fact sheets, PowerPoints, and leadership leaflets. The Training strategy includes modules suitable for anyone exploring their potential as activists, designed to guide participants in developing a passion for and skills in creating positive change, which are offered according to the four phases.	From Communication Materials the power posters, community posters and picture cards from various phases were adapted for use by activists and safe space facilitators. Some materials from this strategy were not adapted for being less relevant to the Rwandan context, such as the card games and comic strips. SASA! Faith communication materials were also adapted. From the Local Activism strategy, community conversations, community dramas and quick chats (including revised healthy relationship chats to have a stronger emphasis on couples) were adapted. Materials from the Media and Advocacy strategy were not adapted for Indashyikirwa, as the programme used other innovations including the training and engagement of opinion leaders. The Training strategy informed the curricula with opinion leaders, women’s space facilitators, couples, and activists. The refresher trainings were not implemented in a phased approach but rather conducted on a more ad hoc basis in response to identified needs.

Most notably, *Indashyikirwa* added an intensive 21-session couple’s curriculum intended to promote healthy relationships, increase couple communication, and decrease male control and violence. This decision was both tactical and strategic. It built on earlier evidence that *SASA!*’s impact on IPV was partially mediated through improved couple relationships, a mechanism of impact not originally emphasised in the *SASA!* theory of change [5]. Additionally, Raising Voices and Center for Domestic Violence Prevention (CEDOVIP) trained community activists slowly over time (inviting them to mini-trainings to learn new content incrementally over two years, then sending them back into the community to share this new knowledge). This strategy was viable in their context because both organisations were co-located in the Kampala communities where they implemented *SASA!*. The desire to implement *Indashyikirwa* at scale in multiple provinces meant that partners needed a strategy to prepare community members for activism that did not require repeatedly convening widely dispersed activists. The couple’s curriculum offered a mechanism to both strengthen the relationship and skills-building element of the programme while condensing the time and costs of preparing community members to serve as mobilisers.

**Figure 1:**
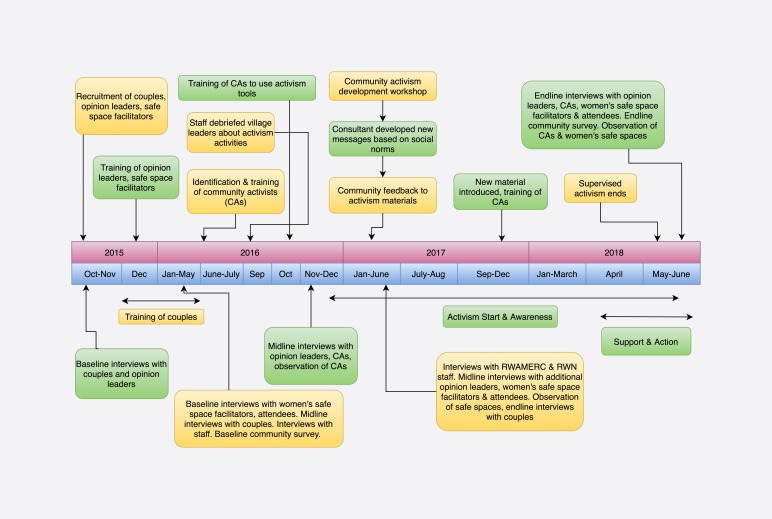
Timeline of intervention and research activities.

**Figure 2:**
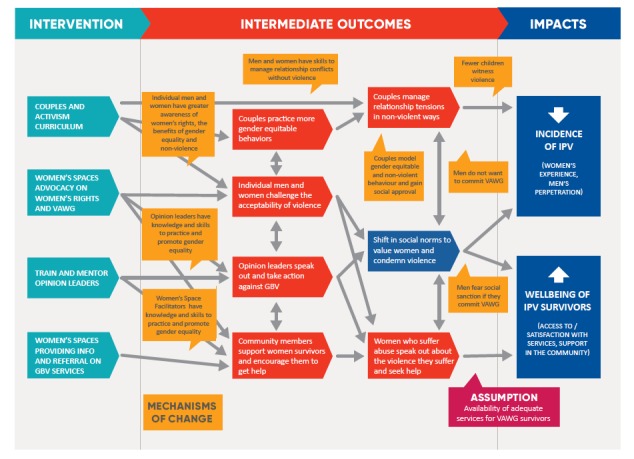
*Indashykirwa* theory of change.

**Figure 1** and **Figure 2** depict the programme’s ultimate implementation and research timeline as well as *Indashyikirwa’s* theory of change. After completing the couple’s programme, individuals who expressed interest and met additional criteria (in terms of literacy, commanding respect in the community, and ability to commit to 2-3 activities per month for 18 months) were invited to attend 15 supplemental sessions on community activism. These sessions emphasised how to use participatory techniques to challenge prevailing beliefs and engage community members around transforming gender norms, balancing power in relationships and interrupting violence. RWAMREC staff both conducted this training and offered ongoing support to CAs through on-site monthly meetings.

To cultivate a supportive environment for community change, RWN likewise trained approximately forty opinion leaders per intervention sector (midlevel administrative units with an average population of 25 000 people) at the beginning of the programme (eg, local government, service providers, religious leaders), and again after local elections replaced some of the incumbants in these positions. To provide additional support for victims, RWN established fourteen “safe space” drop-in centers (one per intervention sector), building on their experience since 1997 running the Polyclinics of Hope for victims of the genocide [14]. The *Indashyikirwa* safe spaces were staffed by trained community volunteers, recruited from the villages where the community activists were active. These volunteers facilitated sessions on IPV and women’s legal rights, and accompanied women who wanted to seek assistance from health, justice and/or social services.

## METHODS

The impact evaluation took the form of a community randomised controlled trial (cRCT), with randomisation at sector level, and two separate evaluation components: (1) a longitudinal cohort of couples who attended the couples intervention and couples recruited in a like manner from comparison communities, interviewed at baseline, 12, and 24 months; and (2) a pair of cross-sectional community surveys implemented near the beginning of the intervention and 24 months later. Results from the community surveys are presented in this paper.

### Design

The community surveys consisted of a household-based random sample of currently partnered women and men residing in villages from the intervention and control sectors (50 men and 50 women surveyed in each of the 28 study sectors). The community survey was repeated 24 months later to assess possible community-level impact of the programme. The *Indashyikirwa* intervention was built on the existing infrastructure of CARE Rwanda’s community-based village savings and loan associations (VSLAs). Prior to the involvement of the What Works evaluation team, the program partners chose 7 districts for implementation of *Indashyikirwa*, spread across Eastern, Northern and Western provinces. These districts were chosen based on examination of the Demographic and Health Survey (DHS) 2010 data to identify areas with high rates of IPV, in combination with a strong presence of CARE’s VSLA program. Office-based rosters of VSLAs were used to identify sectors and cells within these districts with a likely viable concentration of VSLAs from which to recruit couples; these were then verified by an on-the-ground survey.

### Sampling

An independent statistician randomised the sectors deemed viable for programme implementation into intervention and control sectors, with stratification by district. The final number of sectors per district ranged from 2 to 6. Given that there were a fixed number of total sectors available for the study, we estimated the number of interviews per sector required to show a 25% reduction in key outcomes at α = 0.05 and β = 0.80 for outcome indicators with baseline prevalences ranging from 20%-50%. We used intra-cluster correlation coefficients (ICC) estimated conservatively at 0.15 as well as ICC = 0.10 using a formula recommended by Hayes & Bennet for cluster randomised control trials [19]. Based on these estimates, budget, and operational logistics, we planned for 50 interviews each with women and with men per sector, for a target of (28*50) = 1400 interviews, which gave us power to detect a 25% relative reduction in IPV from baseline given a baseline prevalence of IPV = 35% if ICC = 0.15 and given a baseline prevalence of IPV = 30% if ICC = 0.10.

Two cells per sector were selected for implementation of the couples training or recruiting the control cohort based on having a high density of CARE VSLAs. Community surveys were then implemented in these same cells to maximise the possibility of detecting a diffusion effect. It was anticipated that CAs would primarily conduct community-level activities in their own villages or neighboring villages as they were familiar with the context and had access to support systems including other couples who underwent the couples training and their own social networks. One cell per sector was randomly allocated for conducting the women ‘s community survey and the other for conducting the men’s survey. We conducted separate interviews of men and women in order to maximise participant privacy and safety, as suggested by the WHO ethical guidelines [20].

To help control field costs, the community survey was implemented in two contiguous villages within each cell. Because we were most interested in assessing potential community diffusion, site identification began by assessing where participants in the couples training or VSLA control group resided. We then purposively selected 2 adjacent villages with the highest concentrations of cohort participants to create geographic clusters for the purposes of data collection. We interviewed 50 adult women or men in each selected area. The baseline survey successfully collected data from 1399 women and 1400 men living in households within identified community sampling areas, split evenly between intervention and control areas (target 1400 each; one missing woman was ill on the day of her appointment and not replaced). Endline data were collected from 1400 women and 1400 men.

To be eligible for the community survey, individuals had to be between 18 and 49 years old, living with a partner for at least 6 months, and not directly engaged with *Indashyikirwa* as a community activist, trained opinion leader, women’s safe space facilitator, or a member of the Couple’s Cohort.

### Data collection

All data recruitment and data collection was conducted by Laterite Ltd, a private Rwandan research firm. Interviews were conducted in Kinyarwanda via ACASI (audio-enhanced computer self-interviewing) or face-to-face, based on participant preference. Each participant was provided a handheld digital device (in this case, an iPod touch) which presented questions and potential answer choices on a screen while a gender-matched voice read the questions and answer choices aloud to the participant. Participants could then answer by touching the screen. Participants who were not comfortable using ACASI or preferred a face to face interview could opt for this at any point. At baseline 62.1% of women and 85.9% men completed via ACASI, 34.7% of women and 13.0% of men via face-to-face, and 3.2% of women and 1.1% via mixed administration. At endline, 86.1% of women and 90.9% of men completed via ACASI, 12.1% of women and 8.0% of men via face-to-face, and 1.8% of women and 1.1% of men via mixed administration. There was no difference between study arms in the distribution of data collection methods at either time point.

### Ethics and consent

Approval to undertake the study was obtained from the Rwandan National Ethics Committee (REF: No. 340/RNEC/2015) and the National Institute of Statistics Rwanda (REF:0738/2015/10/NISR). Secondary ethics approval was also obtained from the South Africa Medical Research Council (REF: EC033-10/2015). Written consent was obtained from all participants; illiterate participants could have the form read to them by study personnel or a trusted person of their choosing.

Participants were compensated RWF 2000 (approx. US $2.20) for their travel, and a female professional counsellor, organised by the study, was available to support particpants who experienced any distress, with services offered either in person, over the phone, or via referral at a later time.

### Analysis

**Table 2** presents the primary and secondary outcome measures utilised in the community surveys. We utilised multilevel modeling to compare change over time in reported outcomes between the intervention and control groups from baseline to endline. We chose to use an individual-level rather than cluster-level analysis because of the added power that it provides. We included both fixed and random effects to account for the sample design. The fixed effects terms included study arm, data collection wave, and an interaction term for study arm and data collection wave. The district in which data were collected was treated as a fixed effect, and sector (the unit of randomisation) was included as a random effects term. We used generalised linear mixed effects modeling (multilevel model for change) with a Gaussian link function to compare mean scores at end-line for all continuous outcomes [21]. For the binary outcomes, we used generalised linear mixed effects model with a logit link function to compare the effect of the intervention between the two study arms. To test for differences in outcomes over time between participants in the treatment group and participants in the control group, we included an interaction term between treatment (0 = control, 1 = treatment) and wave (0 = baseline, 1 = endline) in all models. Women from the control arm were compared with women from the intervention arm, and men from the control arm with men from the intervention arm. All models included age, education, and asset ownership as covariates. Each model also included the baseline value of the dependent variable aggregated at the sector level as a control variable. There was less than 0.2% missing data on all outcomes and covariates and we used case-wise deletion for individuals who were missing on any of the variables included in our models. Stata 15 (Stata Corp, College Station, TX, USA) was utilised for the data analysis and all comparisons were evaluated at a 5% significance level.

**Table 2:** Outcome measures for quantitative analysis

Construct	Women	Men	How assessed
Primary outcome measures:			
Physical or sexual intimate partner violence, What Works definition, past 12 months	Experience	Perpetration	Adapted WHO violence against women tools; 5 items on physical IPV, 3 items on sexual IPV, covering past 12 months. Answer choices: never, once, a few times, many times. Coded as “yes” per What Works definition is any answer > once or multiple items endorsed.
Acceptability of wife beating (0-5)	Yes	Yes	5 items as per DHS; coded as 1 point for each “Agree” or “Strongly Agree”
Actions to support victims of gender-based violence	Yes	Yes	4 items and summative score
Secondary and exploratory outcome measures:			
Any physical IPV	Experience	Perpetration	An affirmative response on any of the 5 physical IPV items
Any sexual IPV	Experience	Perpetration	An affirmative response on any of the 3 sexual IPV items
Economic abuse with main partner, past 12 months	Experience	Perpetration	3 items, WHO violence against women tools, coded yes for any “once” or higher
Emotional aggression	Experience	N/A	3 items, WHO violence against women tools, coded yes for any “once” or higher
Children witnessing IPV among survivors of IPV	Yes	N/A	Single item on frequency of children witnessing violence against mother
Help seeking among survivors of IPV	Survivors only	N/A	2 items baseline (ever, past year); 1 item at follow-up (past year)

WHO – World Health Organization, IPV – intimate partner vilence, DHS – Demographic Health Survey

In addition to the quantitative components, the team conducted extensive qualitative research including a detailed process evaluation to explore potential pathways of change, and assess programme exposure, implementation delivery, and fidelity [22]. The full qualitative component of the study is detailed elsewhere [23] but methods relevant to the process evaluation are summarised in **Table 3****.** After carefully reading the transcripts, the second author established a preliminary coding structure to thematically analyse the data using NVIVO 11 software. Data was analysed sequentially and qualitative analysis was conducted prior to the quantitative analysis as it took longer for the surveys to be completed and to obtain the data from the data collection agency. The longitudinal qualitative process evaluation was used to help interpret the quantitative results.

**Table 3:** Summary of data sources for process evaluation

Participants	Number Interviewed	Timing	Recruitment Criteria	Scope of Enquiry
Women’s Safe Space Facilitators	3 (1 per province)	May 2016 (after completing programme training and beginning role as facilitators); June 2017; June 2018	1 facilitator per safe space recruited by RWN staff	Motivations to be facilitators and their impressions of the programme training; Perceived impact of the safe spaces and the support they receive as facilitators
Community Activists	12 = 6 men and 6 women (2 men and 2 women per province)	November 2016 (after completing activism training and beginning activist activities); May 2018	Activists who had not participated in couples’ interviews recruited by RWAMREC staff	Impressions of the activism training, what motivated them to continue as activists, what they had been doing recently as activists, and whether they faced any challenges
RWAMREC and RWN Staff	**16** = 9 RWAMREC staff and 6 RWN staff and 1 CARE Staff across intervention sectors	May 2016 (after delivering the couples, opinion leaders and women’s space facilitators curriculum); May 2017; September 2018	Diversity of field officers and field supervisors across various intervention sectors and districts	Successes and lessons learned from facilitating curricula with opinion leaders and women’s safe space facilitators and engaging opinion leaders and operating the women’s safe spaces (RWN staff), and from facilitating couples’ curriculum and supporting community activism with trained partners of couples (RWAMREC staff)

RWAMREC – Rwanda Men’s Resource Centre, RWN – Rwanda Women’s Network

## RESULTS

Descriptive data are presented separately for women and men in **Table 4** and **Table 5**. There were no statistically significant differences at baseline between study arms on sociodemographic measures or potential confounding variables with one exception: a higher proportion of men at baseline reported problematic alcohol use in the intervention communities (15% vs 9%) as compared to the control communities.

**Table 4:** Descriptive data for female participants

	Baseline					Endline				
	**Intervention**		**Control**			**Intervention**		**Control**		
**Variable*s***	**N**	**% or mean**	**N**	**% or mean**	***P-*value**	**N**	**% or mean**	**N**	**% or mean**	***P-*value**
Age (years):										
≤25	115	16.5%	102	14.6%	0.2	124	17.7%	109	15.6%	0.3
26-30	181	25.9%	169	24.1%		166	23.7%	173	24.7%	
31-35	180	25.8%	167	23.9%		191	27.3%	172	24.6%	
36-40	121	17.3%	140	20.0%		135	19.3%	155	22.1%	
≥41	102	14.6%	122	17.4%		84	12.0%	91	13.0%	
Education:										
None	102	15.0%	138	20.1%	0.2	128	18.0%	129	18.5%	0.7
Primary	461	66.0%	450	64.0%		451	64.4%	429	61.4%	
Secondary or above	135	19.0%	112	16.0%		121	17.3%	141	20.2%	
Marital status:										
Married	424	61.0%	404	58.0%	0.6	391	55.9%	363	51.9%	0.4
Living as if married	274	39.0%	296	42.0%		309	44.1%	337	48.1%	
Polygamy:										
Yes	62	9.0%	80	11.0%	0.3	81	12.0%	66	9.4%	0.6
No	514	74.0%	534	76.0%		423	60.4%	468	66.9%	
Do not know	123	18.0%	86	12.0%		196	28.0%	166	23.7%	
Children:										
None	39	5.6%	37	5.3%	0.5	45	6.4%	47	6.7%	0.5
One	100	14.3%	98	14.0%		130	18.6%	115	16.4%	
Two	164	23.5%	157	22.4%		161	23.0%	155	22.1%	
Three	163	23.3%	157	22.4%		149	21.3%	153	21.9%	
Four or more	233	33.3%	251	35.9%		214	30.6%	230	32.9%	
Income:										
No cash	265	37.9%	242	34.6%	0.1	359	51.3%	323	46.0%	0.5
<RWF5000	262	37.5%	248	35.4%		204	29.1%	239	34.2%	
RWF5001-10 000	97	13.9%	110	15.7%		77	11.0%	83	11.9%	
>RWF10 000	75	10.7%	100	14.3%		60	8.6%	54	7.7%	
Debt in previous month:										
No debt	134	19.2%	133	19.0%	0.5	136	19.4%	133	19.0%	0.3
<RWF1000-2000	122	17.5%	118	16.9%		190	27.1%	206	29.4%	
RWF2001-10 000	191	27.4%	167	23.9%		151	21.6%	164	23.4%	
>RWF10 000	251	36.0%	282	40.3%		223	31.9%	197	28.1%	
Land ownership:										
Own land	149	21.3%	192	27.4%	0.4	161	23.0%	165	23.6%	0.3
Rent land	239	34.2%	194	27.7%		239	34.1%	247	35.3%	
Own and rent	245	35.1%	250	35.7%		188	26.9%	196	28.0%	
Do not own or rent	66	9.4%	64	9.1%		112	16.0%	92	13.1%	
Home ownership	595	85.1%	597	85.4%	0.9	589	84.0%	567	81.0%	0.3
Asset ownership:										
Radio	397	57.0%	336	48.0%	0.1	338	48.0%	303	43.0%	0.4
Electricity	158	23.0%	96	14.0%	0.2	227	32.0%	174	25.0%	0.2
Bicycle	167	24.0%	183	26.0%	0.7	171	24.0%	175	25.0%	0.9
Cellphone	504	72.0%	456	65.0%	0.1	483	69.0%	457	65.0%	0.3
Weighted asset score		7.1		6.9	0.3		7.2		7.4	0.3
Earning disparity:										
About same	69	6.7%	96	5.4%	1.0	64	9.2%	87	12.4%	0.5
All work together	327	36.5%	324	34.4%		239	34.2%	281	40.1%	
Husband more	47	47.0%	38	46.0%		300	43.0%	283	40.4%	
Wife more	255	9.9%	240	13.8%		95	13.6%	49	7.0%	
Hunger score		4.9		4.9	0.8		5.0		5.0	0.6
Alcohol use:										
Seen male partner drunk	354	68.0%	358	71.0%	0.6	386	75.0%	339	69.0%	0.3
Woman’s alcohol use:										
None	548	78.4%	570	81.4%	0.4	616	88.0%	610	87.1%	0.7
Any	101	14.5%	91	13.0%		55	7.9%	57	8.1%	
Alcohol problem/issue	50	7.2%	39	5.6%		29	4.1%	33	4.7%	
VSLA membership:										
I belong	230	33.0%	214	31.0%	0.4	254	36.3%	203	29.0%	0.1
Spouse belongs	119	17.0%	104	15.0%		108	15.4%	125	17.9%	
Both	199	29.0%	221	32.0%		185	26.4%	181	25.9%	
None	150	21.0%	161	23.0%		153	21.9%	191	27.3%	
Previous experience of IPV:										
Physical	25	27.0%	23	20.0%	0.3	37	40.0%	27	30.0%	0.1
Sexual	56	8.0%	52	7.0%	0.8	39	41.0%	21	22.0%	0.0
Forced first sexual experience	180	26.0%	173	25.0%	0.7	197	28.0%	173	25.0%	0.3

CI – confidence interval, VSLA **–** village savings and loan associations, IPV – intimate partner violence

**Table 5:** Descriptive data for male participants

	Baseline					Endline				
	**Intervention**		**Control**			**Intervention**		**Control**		
**Variables**	**N**	**% or mean**	**N**	**% or mean**	***P*-value**	**N**	**% or mean**	**N**	**% or mean**	***P*-value**
Age (years):										
≤25	55	7.9%	47	6.7%	0.3	41	5.9%	58	8.3%	0.4
26-30	138	19.7%	174	24.9%		134	19.1%	135	19.3%	
31-35	195	27.9%	188	26.9%		180	25.7%	199	28.4%	
36-40	134	19.1%	154	22.0%		181	25.9%	138	19.7%	
≥4	178	25.4%	137	19.6%		164	23.4%	170	24.3%	
Education:										
None	116	17.0%	125	18.0%	0.7	139	19.9%	128	18.3%	0.6
Primary	460	66.0%	453	65.0%		451	64.4%	458	65.5%	
Secondary or above	124	18.0%	122	17.0%		110	15.7%	113	16.2%	
Marital status:										
Married	461	66.0%	424	69.0%	0.3	418	59.7%	376	53.7%	0.3
Living as if married	239	34.0%	276	39.0%		282	40.3%	324	46.3%	
Polygamy:										
Yes	32	4.6%	41	5.9%	0.2	45	6.4%	40	5.7%	0.6
No	668	95.4%	659	94.1%		655	93.6%	660	94.3%	
Children:										
None	33	4.7%	39	5.6%	0.5	46	6.6%	55	7.9%	0.4
One	109	15.6%	90	12.9%		119	17.0%	119	17.0%	
Two	142	20.3%	173	24.7%		149	21.3%	147	21.0%	
Three	130	18.6%	139	19.9%		144	20.6%	150	21.5%	
Four or more	286	40.9%	259	37.0%		242	34.6%	228	32.6%	
Income:										
No cash	220	31.0%	238	34.0%	0.8	207	29.6%	187	26.7%	0.6
<RWF5000	273	39.0%	268	38.3%		281	40.1%	282	40.3%	
RWF5001-10 000	108	15.4%	91	13.0%		122	17.4%	132	18.9%	
>RWF10 000	99	14.1%	103	14.7%		90	12.9%	99	14.1%	
Debt in previous month:										
No debt	109	15.6%	109	15.6%	0.4	88	12.6%	133	19.0%	0.0
<RWF1000-2000	211	30.1%	235	33.6%		247	35.3%	243	34.8%	
RWF2001-10 000	175	25.0%	161	23.0%		149	21.3%	128	18.3%	
>RWF10 000	205	29.3%	195	27.9%		216	30.9%	195	27.9%	
Land ownership:										
Own land	163	23.3%	183	26.0%	0.1	139	19.9%	158	22.6%	0.8
Rent land	222	31.8%	239	34.1%		249	35.6%	237	33.9%	
Own and rent	251	35.9%	209	29.9%		235	33.6%	214	30.6%	
Do not own or rent	63	9.0%	69	9.9%		76	10.9%	91	13.0%	
Home ownership	613	88.0%	603	86.0%	0.5	621	88.0%	589	84.0%	0.1
Asset ownership:										
Radio	432	61.7%	383	54.7%	0.1	396	57.0%	388	55.0%	0.8
Electricity	140	20.0%	115	16.0%	0.6	197	28.0%	210	30.0%	0.8
Bicycle	244	35.0%	210	30.0%	0.5	220	31.0%	182	26.0%	0.4
Cell phone	506	72.0%	496	71.0%	0.7	522	75.0%	494	71.0%	0.2
Weighted Asset Score		7.5		6.9	0.3		7.6		7.39	0.3
Earning disparity:										
About same	193	28.0%	172	24.6%	0.4	160	22.9%	152	21.8%	0.6
All work together	83	6.2%	74	7.5%		71	10.1%	70	10.0%	
Husband more	43	11.9%	52	10.6%		97	13.9%	87	12.5%	
Wife more	378	54.2%	400	57.3%		372	53.1%	390	55.8%	
Hunger score		5.0		5.1	0.5		4.9		5.0	0.6
Alcohol use:										
None	393	56.1%	484	69.1%	<0.001	407	58.1%	429	61.3%	0.3
Any	205	29.3%	156	22.3%		180	25.7%	187	26.7%	
Alcohol problem/issue	102	14.6%	60	8.6%		113	16.1%	84	12.0%	
VSLA membership:										
I belong	186	27.0%	189	27.0%	0.6	144	20.6%	144	20.6%	0.8
Spouse belongs	140	20.0%	134	19.0%		158	22.6%	184	26.3%	
Both	233	33.0%	214	31.0%		256	36.6%	215	30.7%	
None	140	20.0%	163	23.0%		142	20.3%	157	22.4%	
Physical IPV against previous partner	76	10.9%	59	8.0%	0.2	69	25.0%	43	16.0%	0.1
Sexual IPV against previous partner	27	4.0%	30	4.0%	0.7	36	12.0%	31	10.0%	0.4
Witnessed mothers' abuse as a child	324	46.0%	289	41.0%	0.2	347	50.0%	322	46.0%	0.4
Physically abused as a child:										
Never/sometimes	491	70.0%	460	66.0%	0.2	431	61.6%	440	62.9%	0.8
Often	132	19.0%	151	22.0%		160	22.9%	147	21.0%	
Very often	77	11.0%	89	13.0%		109	15.6%	113	16.1%	

VSLA **–** village savings and loan associations, IPV –intimate partner violence

Women ranged from 15 to 49 years and were evenly distributed across arms in terms of age, with roughly 16% of women less than or equal to 25 years old, and an equal proportion (15%), 41 years or older, at both baseline and endline. Men ranged in age from 19 to 51 years with roughly half between 26 and 35 years old in both intervention and control communities. Two thirds of both female and male participants had completed primary school and were formally married, in both cross-sectional samples.

### Primary outcomes

Multivariate results for women and men are presented in **Table 6** and **Table 7**. Among women, there was no significant difference between participants in the intervention and control communities in change over time of experiencing physical and/or sexual IPV from a current male partner in the past 12 months. The adjusted odds ratio (aOR) for the intervention impact on self-reported experience of IPV was aOR = 1.25 (95% CI = 0.92-1.70, *P* = 0.16). Likewise, there was no difference in change over time in self-reported rates of perpetration of physical and/or sexual IPV among male participants in the treatment and control groups (aOR = 1.02; 95% CI = 0.72-1.45, *P* = 0.89).

**Table 7:** Multivariate results for all men*

	Study arm	Baseline % or mean	Endline % or mean	aOR/	95% CI		*P*-value
coeff	lower	upper
**Primary outcomes:**							
Physical and/or sexual intimate partner violence with main partner	Control	19.7%	21.4%				
	Intervention	31.7%	34.7%	1.02	0.72	1.45	0.89
Acceptability of wife beating (number of reasons endorsed as justifications, range 0-5)	Control	0.92	0.91				
	Intervention	0.92	1.03	0.09	-0.10	0.29	0.34
Actions to support victims of gender-based violence or combat gender-based violence (range 0-12)	Control	7.72	7.58				
	Intervention	7.60	7.29	-0.13	-0.65	0.40	0.64
**Secondary outcomes:**							
Sources of information on IPV and number of times heard (range 0-36)	Control	20.4	21.9				
	Intervention	21.3	22.0	-0.70	-2.15	0.76	0.35
**Other outcomes measures (exploratory):**							
Physical intimate partner violence, main partnership	Control	22.5%	24.1%				
	Intervention	30.2%	35.5%	1.14	0.81	1.61	0.44
Forced or coerced sex with main partner	Control	16.7%	17.3%				
	Intervention	27.3%	29.8%	1.08	0.74	1.55	0.70
Economic abuse with main partner	Control	33.2%	34.1%				
	Intervention	34.6%	40.9%	1.23	0.90	1.69	0.19
Children in household witnessing IPV	Control	31.7%	28.7%				
(N = 470 baseline, N = 476 household with children under 18, who reported physical or sexual IPV, and did not respond “don't know” regarding child witnessing)							
	Intervention	32.8%	40.1%	1.66	0.93	2.94	0.09
Support for women working outside the home (range -4 to +4)	Control	0.97	0.84				
	Intervention	0.96	0.74	-0.12	-0.43	0.19	0.45

aOR – adjusted odds ratio, CI – confidence interval, IPV – intimate partner violence

*All models control for age, education, and asset score.

Similarly, there were no significant difference in scores for the acceptability of wife beating at a population level among either female (β = 0.04, 95% CI = -0.23-0.31, *P* = 0.77) or male (β = 0.09, 95% CI = -0.10-0.29, *P* = 0.34) participants in the control or treatment groups over time. Neither was there evidence of an increase in the support offered to victims of gender-based violence in the intervention or control sectors among female β = -0.09 (95% CI = -0.61-0.44, *P* = 0.75) and male β = -0.13 (95% CI = -0.65-0.40, *P* = 0.64) participants.

Thus, we were unable to reject the null hypothesis of no intervention impact.

### Secondary outcomes

There were no significant difference in scores for the sources of information on IPV and number of times heard at a population level among either female (β = -0.04, 95% CI = -1.41-1.33, *P* = 0.96) or male (β = -0.70, 95% CI = -2.15-0.76, *P* = 0.35) participants in the control or treatment groups over time. Among women, there were also no detectable differences in help seeking among victims living in the intervention compared to control communities (aOR = 1.15; 95% CI = 0.79-1.68 *P* = 0.46).

### Exploratory outcomes

We found no evidence of a significant difference among women in the intervention and control communities in change over time in the experience of physical (aOR = 1.27; 95% CI = 0.93-1.73, *P* = 0.13) or emotional IPV (aOR = 1.16; 95% CI = 0.81-1.66, *P* = 0.43) from a current male partner in the past 12 months. Among women, there was weak evidence of potential increased reporting of sexual IPV over time for the intervention group compared to the control group (aOR = 1.35, 95% CI = 0.99-1.82, *P* = 0.06). There was no significant difference, however, in the reporting of perpetration of sexual IPV by male (aOR = 1.08, 95% CI = 0.74-1.55, *P* = 0.70) participants in the control vs treatment groups over time. Women in the treatment group likewise reported more economic violence over time than those in the control group (aOR = 1.36, 95% CI = 1.00-1.85, *P* = 0.05). We did not find any evidence of an increase in the reporting of perpetration of economic IPV between men in the treatment and control groups over time (aOR = 1.23, 95% CI = 0.90-1.69, *P* = 0.19).

We also tested for differences among participants in the control and treatment groups for a range of other exploratory outcomes including support for women’s participation in the laborforce, children witnessing IPV, and change in strategies used to support individuals experiencing IPV. We did not find any statistically significant differences in any of these outcomes, whether reported by female or male participants.

Despite the lack of a measurable community-level effect of the activism activities on IPV, both survey and qualitative data suggest that the women’s safe spaces were generally well utilised and regarded by communities. **Table 8** shows the number of people who were attending the women’s spaces and accessing services. The fact that 92.8% of women and 96.2% of men were aware of the service and were willing to recommend it to others speaks to the excellent reputation of the safe spaces across intervention communities. Moreover, the majority of those who reported attending activities or seeking services at the women’s spaces did so more than once.

**Table 8:** Findings from women’s safe spaces

	All respondents					Those who had heard of women's spaces				
	**Women**		**Men**			**Women**		**Men**		
**Variables**	**N**	***%***	**N**	***%***	***P*-value**	**N**	***%***	**N**	***%***	***P*-value**
Have you ever heard about the women space? (out of N = 700 women and N = 700 men interviewed in intervention communities)										
	483	69.00%	497	71.00%	0.63		N/A		N/A	N/A
Have you ever been involved in the activities of the women’s space?										
Yes, once	86	12.30%	93	13.30%	0.44	100	20.70%	86	17.30%	0.07
Yes, twice	89	12.70%	77	11.00%		89	18.40%	77	15.50%	
Yes, more than twice	170	24.30%	162	23.10%		170	35.10%	162	32.50%	
Total	345	49.30%	332	47.40%		358	74.20%	325	65.30%	
Have you sought assistance from the women’s space for problems you were having?										
Yes, once	74	10.60%	78	11.10%	0.47	74	15.30%	78	15.70%	0.44
Yes, twice	31	4.40%	35	5.00%		31	6.40%	35	7.00%	
Yes, more than twice	38	5.40%	53	7.60%		38	7.90%	53	10.60%	
Total	143	20.40%	166	23.70%		142	29.50%	166	33.30%	
Are you aware of anyone else who has sought service from the women’s space?										
	253	36.10%	303	43.30%	0.08	253	52.40%	303	61.00%	0.01
Would you advise other women to seek assistance from the women’s space?										
	450	64.30%	479	68.40%	0.34	448	92.80%	478	96.20%	0.06

### Process evaluation findings

The process evaluation data suggests several potential reasons why the activism component of *Indashyikirwa* failed to reduce IPV at a community level, specifically problems applying the *SASA!’s* style of “informal activism” to the Rwandan context; unanticipated delays in rolling out the activism component of the programme; and confusion over the notion of “phasing” – a concept central to the *SASA!* theory of change.

#### Challenges in cultural adaptation

A core concept of the *SASA!* approach to community mobilisation is the notion of “informal” activism – engaging community members in conversation where they congregate: at moto stands, local repair shops or local markets. Process evaluation data suggest that this type of “informal activism” did not translate easily to the Rwandan setting, where more formal settings are the norm [13]. Indeed, many CAs and program staff reflected on how community members did not feel comfortable discussing intimate matters in public:

“Rwandans are not used to discuss their issue in public. You need to choose a safer place to help people gain their trust. On the side of the road or at a market it will be hard.” (RWAMREC Field Supervisor, Western Province).

CAs found that participants preferred to enagage in more formal environments. Because of this, activists used existing forums, such as parents evening forums, *umuganda*, and VSLA groups to deliver their messages [23]. This likely limited the diffusion of program content, especially if the same individuals attended these events over time. Indeed, one supervisor expressed concern that their monitoring data likely double counted beneficiaries since CAs returned multiple times to the same community forms:

“We don’t know how many people we met, how many people got the message in a month. We only have these VSLs, village meetings, we don’t have many opportunities…One activist may go to a VSL and meet 15 people, and the next day another activist goes to the same VSL. We meet the same people because we don’t have enough opportunities to meet people.” (RWAMREC Field Supervisor, Eastern Province)

Such forums can have a large amount of attendees and as a result, often demand a more didactic style of communication than the interactive engagement anticipated by the *SASA!* model. CAs observed that when facilitating activism at government led initiatives, they were frequently only afforded 5 minutes or less by opinion leaders at the end of a meeting to impart their message. This challenge was helpfully addressed through RWAMREC staff emphasizing to local leaders the importance of CAs having more time to facilitate their activities, and through leaders increasingly recognizing the value of the activism efforts.

Indeed, there was much discussion on behalf of CAs and staff describing how the participatory approaches to facilitation and dialogue anticipated by *SASA!* were a novel approach for many CAS, and it took effort to help them feel more comfortable with and have opportunities for this model.

“The [CAs] do not do informal activism. We push them to go to markets, churches, bus stations, but they are shy. They don’t dare go there. When we ask local leaders or pastors, they say we have these opportunities, but when we ask community acitivists to go there, they are still shy. I think this is related to the new approach because Rwandans are not familiar with this kind of thing. At first people were scared to talk in public but there is improvement, slowly.” (RWAMREC Field Supervisor, Eastern Province).

Although this engagement eventually evolved into opportunities for participatory discussions with smaller groups, this process took considerable time, which likely compromised the programme’s potential impact. It would have been beneficial to have modified the *SASA!* model and piloted the cultural modifications before conducting a cRCT.

#### Shortened implementation

Data from monitoring interviews with project managers and field staff, and observations of CAs and women’s space facilitators, likewise confirm that the time available for activism was trunctated, likely limiting the programme’s ability to achieve its objectives. Because of the time required to finalise the *Indashyikirwa* design, pilot and implement the couple’s curricula, and adapt *SASA!* activist materials to the Rwandan setting, *Indashyikirwa* only had 1.5 years available for activism (November 2016-June 2018). The *SASA!* fidelity brief suggests that a minimum of 3 to 5 years of community mobilisation is necessary to shift norms and reduce IPV [18].

Programme implementation also encountered various delays that ate into the time available for community mobilisation. Community activists were delayed in the first instance because not all village leaders were initially briefed about the program or involved in the opinion leader training [23]. Introductions to the programme were originally held at the sector level, rather than the cell or village level; this meant that many village heads had not been briefed when activists began their activities. This proved especially problematic because village heads served as critical gatekeepers to many of the formal community structures, such as domestic violence committees and parenting forums, that CAs initially relied upon. Without support from the village head, community activists did not feel comfortable or able to facilitate activism. As one field officer reflected:

The need to gain support from village leaders necessitated an additional (and unanticipated) round of project introductions by program staff throughout the fourteen sectors before activism activities could get under way.

Program partners universally reported that the activism component of *Indashyikirwa* was far too rushed [23] and that community members did not have adequate time to internalise and apply the more advanced concepts of *SASA!* focused on encouraging norm change and taking action against violence.

“I don’t think we have enough time for activism. It is not long enough. Skipping from this phase to the other, I think it should require a certain long time. If we are copying SASA!, we are a bit squeezed for time.” (RWAMREC Field Officer)

Some activists lamented not having a diversity of materials earlier on:

“The images are not enough. There are some groups where we finished all of the images and now, we go back with them again.” (Male Activist 01 Western Province)

“Also, in this village, they now know many things and sometimes when we are going to discuss on a certain image, they say: “no, it has been a long time since we studied that image, let us discuss about that other one.” (Male Activist 01 Eastern Province)

Despite these challenges, activists noted the changes in participants’ understanding of positive and negative types of power and different types of IPV (physical, sexual, emotional and economic) during the start and awareness phases, as this content was emphasised through the activism materials and messages:

“What I like to tell the community people about forced sexual intercourse is that, that is not good. For example, when a husband came home being drunk, he used to force his wife to go to bed for sexual intercourse. We have talked with women, now many women have understood what violence is.” (Male Activist 01 Western Province)

### Phasing and diffusion

In addition to cultural discordance and delays, the opportunity for deeper transformation was further complicated by lack of clarity around the notion of phasing—a core concept of *SASA!’s* theory of change. Each phase of *SASA!* focuses on a different outcome: start (knowledge), awareness (attitude), support (skills), action (behavior). In *SASA!,* monitoring and evaluation tools are used to assess progress through the phases and at the end of each phase, a Rapid Assessment Survey helps determine if “the community is ready to move to the next phase [18].”

Phased programming was a new and unfamiliar concept for the partners, and it was difficult to anticipate both when to move onto the next phase, as well as the effort required to do so [23]. There was a lack of consensus among field officers about when and how to encourage activists to move on in their activities. Notably, field officers did not receive sufficient training in the phased aspect of the programming and delivery of relevant messages:

“Field staff did not have a clear map that Phase I should go for this period, and then Phase 2 should be this long. Phase 1 and 2 was way longer and we were at the end of the project and we had to combine Phase 3 and 4. We did not know how to handle the phases and it was not mapped during our initial training”. (RWAMERC Field Supervisor)

“It was not easy for staff to get the hang of how the activism was meant to happen. Moving from one phase to another…One of the things was depending on the understanding of the field officers, we had certain places where activism was working better than in other places…Some of them did not know what to do with the posters, and when to use them.” (CARE Field Officer)

This uncertainty may have contributed to delays in encouraging and reinforcing activities around supporting victims and taking action to prevent violence—both important secondary outcomes for *Indashyikirwa*’s community programme.

## DISCUSSION

This study assessed the impact of the community activism component of the *Indashyikirwa* program on the prevention of IPV in a community sample. The process evaluation suggests that the overall lack of effect may have been due to implementation challenges. Even under the best conditions, it is rare for programmes to demonstrate significant reductions in IPV at a population level; only three trials (*SASA!*, SHARE, and the COMBAT in Ghana) have done so thus far [3,6,7].

The *SASA!* fidelity brief suggests that 3-5 years of activist activity is required for phased implementation and activism to cover the required content necessary to shift norms and effect behavior change at a population level [18]. A core feature of *SASA!* is that it is designed to be implemented in phases, with content and support material rolled out over time. In *Indashyikirwa*, the Start and Awareness phase of *SASA*! were condensed into one (lasting roughly 16 months) and the third and fourth phases were similarly combined, with the materials for the Support and Action phases only made available in April 2018, 3 months before the evaluation endline. Most activists therefore relied largely on a few materials from the “start” and “awareness” phase of *SASA!*, which focused on introducing the four types of power (a key *SASA!* concept) and awareness of different types of violence.

While the time squeeze was due in part to avoidable delays, it also was a function of the time it takes to develop and pilot new interventions and adapt existing ones to new cultural settings. In our experience, donors currently underestimate the time it takes to undertake these activities responsibly and with rigor. To ensure project success, program planners and donors must allow sufficient time to accommodate all phases of program design and implementation as well as unexpected (but typical) programme delays. Future programmes of this complexity should be funded and evaluated over 5 to 7 years to ensure a fair assessment of project impact.

Our experience with *Indashyikirwa* further suggests that evidence based practices (EBP) need to be modified to suit the cultural needs of new communities, especially regarding the complexity of community processes [23-25]. The informal activism that was successful in urban Uganda did not easily translate easily to the highly organised structure of rural Rwanda. In their evaluation of the *SASA!* model, Starmann et al. [5] found that interpersonal communication was critical to the overall success of *SASA!* Rwandan CA’s initial reliance on formal structures, such as *umuganda*, limited opportunities for reflection and interpersonal exchange. Many staff and activists nonetheless noted that home visits and couple to couple sharing emerged as highly successful strategies for diffusing programme content over time [23]. Thoughtful adaptation of programmes requires adjusting the *means* to achieve programme ends, rather than slavishly adhering to a given strategy.

The *Indashyikirwa* experience likewise raises questions about the degree to which phasing should be taken as central to the success of community mobilisation approaches to violence reduction. In *SASA!*, phasing serves two purposes: it allows activists to be trained in stages and ensures that community members grasp key concepts before moving onto new ones. It is yet unclear whether the benefits of allowing knowledge and attitude change to consolidate prior to encouraging action outweigh the potential confusion and delay caused by postponing action. Indeed, the designers of *SASA!* have modified this element in their newly released programme known as *SASA Together* (L. Michau, personal communication, October 25, 2019). *SASA Together* retains the notion of phasing but identifies a Knowledge, Feeling and Action goal for each phase. At the very least, the Rwandan experience highlights the importance of thoroughly orienting field staff to the logic and mechanics of phasing so that confusion does not hinder progress.

Our findings also raise the possibility that as implemented, the *Indashyikirwa* activist programme may have increased reporting of IPV among women, especially forced sex and economic violence by an intimate partner. Although, none of the point estimates achieved our pre-specificed level of significance of *P* < 0.05, the estimates were uniformly above 1 and there was marginal evidence that reporting of sexual (aOR = 1.35, 95% CI = 0.99-1.82, *P* = 0.06) and economic violence (aOR = 1.36, 95% CI = 1.00-1.85, *P* = 0.05) increased over time. Longitudinal analysis of the qualitative couple data in intervention communities indicates that both women and men were more likely to acknowledge sexual violence in their relationships over time [26], and higher levels of reported IPV overall would be consistent with the programmes’achieved emphasis on awareness raising, vs later stages of the *SASA!* change model.

In conclusion, community mobilisation strategies as a means to reduce levels of IPV deserve further investigation in rural Rwanda and in other rural settings. Consideration should be given up front into how best to encourage interpersonal reflection and collective action to transform norms and practices on gender, power and violence. Implementing agencies and donors should use the *Indashyikirwa* community component as an object lesson for the importance of cultural adapatation, adequate training and length of implementation.

As with all projects, this impact evaluation has a number of limitations. First, all measures rely on self-report which means they are subject to under-reporting and disclosure bias. There may also be social desirability bias around participants wanting to report favorably on an intervention they clearly valued or wanting to emphasise what they learned. We attempted to mitigate this strategy through offering the use of ACASI data collection. Evidence suggests that ACASI encourages more truthful and forthcoming reporting for stigmatised topics [27,28]. We also attempted to mitigate social desirability effects by using field researchers who were external to the program, specially trained in gender sensitivity, and emphasising the confidentiality of all answers.

The evaluation also had limited means to evaluate individual-level exposure to project activities. We chose to ask participants about past-year exposure to IPV messaging via a range of interpersonal, social, and communication channels at both baseline and endline, hypothesising that *Indashyikirwa’s* sector-level activites would increase overall exposure. However, self-reported exposure to messaging was very high at baseline, leaving little room for movement, and the use of ACASI made it difficult at endline to assess exposure to messaging originating from *Indashyikirwa* vs other sources, although we do know that exposure to the women’s safe spaces did not impact reports of IPV (data not shown). We strongly recommend that when assessing tradeoffs in data collection strategies, future studies find ways to both quantitively and qualitatively explore individual-level exposure to programme-specific activites and content of messages received. Such data will be valuable for both process evaluation and interpetation of impact evaluations, especially in the case of null findings at the community-level.

## CONCLUSION

Although this study did not demonstrate violence reduction at the community level, it affirms the importance of program design and careful adaptation of EBPs to ensure fidelity and cultural appropriateness, and the importance of budgeting sufficient time for mobilisation activities before undertaking endline assessment. Failure to do so undermines the value of investing in rigorous randomised evaluations. This study also reinforces the value of embedded process evaluations, especially around the complex phenomenon of community activism and organised diffusion. We strongly recommended that all impact evaluations of violence interventions include both quantitative and qualitative components, preferably collecting both types of data repeatedly when possible.

## 

**Table:** **Table 6.** Multivariate results for all women*

	Study arm	Baseline % or mean	Endline % or mean	aOR/	95% CI		*P*-value
**coeff**	**lower**	**upper**
**Primary outcomes:**							
Physical and/or sexual intimate partner violence with main partner	Control	50.9%	49.7%				
	Intervention	58.9%	63.1%	1.25	0.92	1.70	0.16
Acceptability of wife beating (number of reasons endorsed as justifications, range 0-5)	Control	2.40	2.50				
	Intervention	2.00	2.20	0.04	-0.23	0.31	0.77
Actions to support victims of gender-based violence or combat gender-based violence (range 0-12)	Control	7.10	6.70				
	Intervention	7.10	6.60	-0.09	-0.61	0.44	0.75
**Secondary outcomes:**							
Sources of information on IPV and number of times heard (range 0 - 36)	Control	19.90	20.20				
	Intervention	21.00	21.10	-0.04	-1.41	1.33	0.96
Help seeking among survivors of IPV (N = 872 baseline, N = 933 endline)	Control	54.3%	51.8%				
	Intervention	55.1%	56.7%	1.15	0.79	1.68	0.46
**Other outcomes measures (exploratory):**							
Physical intimate partner violence, main partnership	Control	40.9%	41.9%				
	Intervention	49.6%	56.7%	1.27	0.93	1.73	0.13
Forced or coerced sex with main partner	Control	46.0%	43.8%				
	Intervention	50.5%	55.6%	1.35	0.99	1.82	0.06
Economic abuse with main partner	Control	49.1%	53.6%				
	Intervention	52.1%	64.0%	1.36	1.00	1.85	0.05
Emotional aggression with main partner	Control	71.9%	73.8%				
	Intervention	78.5%	82.3%	1.16	0.81	1.66	0.43
Children in household witnessing IPV	Control	46.5%	47.3%				
(N = 798 baseline, N = 786 control household with children under 18, who reported physical or sexual IPV, and did not respond “don't know” regarding child witnessing)							
	Intervention	46.2%	54.6%	1.29	0.86	1.94	0.22
Support for women working outside the home (range -4 to +4)	Control	1.33	1.42				
	Intervention	1.24	1.25	-0.09	-0.40	0.21	0.54
Change in strategies used to address IPV (range 0-12)	Control	3.17	2.98				
	Intervention	3.23	3.56	0.50	-0.13	1.13	0.12

aOR – adjusted odds ratio, CI – confidence interval, IPV – intimate partner violence

*All models control for age, education, and asset score.
